# Eccentric Cycling in the Real Life: A Case Report Exploring Riding Downhill with a Brakeless Bicycle

**DOI:** 10.5114/jhk/193941

**Published:** 2024-12-19

**Authors:** Nicolas Babault, Carole Cometti

**Affiliations:** 1 INSERM UMR1093-CAPS, Université de Bourgogne, UFR des Sciences du Sport, Dijon, France.; 2 Centre d’Expertise de la Performance, Université de Bourgogne, UFR des Sciences du Sport, Dijon, France.

**Keywords:** muscle activation, lengthening, isometric, fixed-gear, biomechanics

## Abstract

This case study aimed to explore neuromuscular strategies used by a cyclist to control the speed while riding downhill a mountain with a brakeless, fixed-gear bicycle. Accelerations of the pedals and electromyographic activity of four lower limb muscles were registered to determine muscle activation during two decelerating strategies. Eccentric cycling was mostly used to control the bicycle speed with short (536 ± 51 ms) and low-intensity contractions. Isometric pedaling cycles were more efficient for decelerations with long (1,092 ± 281 ms) and intensive contractions. Isometric muscle activation was 122, 31, 25 and 44% greater than eccentric for vastus lateralis, biceps femoris, gastrocnemius lateralis and tibialis anterior muscle, respectively. This study suggests specific activation patterns to help the practitioner for safety rides, but that could have implications for rehabilitation purposes.

## Introduction

Eccentric exercise (defined as the lengthening of the muscle-tendon complex) is often used during rehabilitation as it elicits a lower cardiorespiratory demand and perceived effort than conventional exercise (Clos et al., 2021). It is often performed using indoor ergocycles to involve large muscle mass and complex coordination particularly amongst the knee extensor and calf muscles. Eccentric cycling also exists in real life settings while performing fixed-gear cycling. This form of urban cycling consists of riding track bicycles on roads. With only one speed, no free-wheel and generally brakeless, decelerations are a matter of lower limb force with muscles acting in isometric (no changes of the length of the muscle-tendon complex) or eccentric contractions to block or slow down the rear wheel, respectively. The efficiency of these two braking strategies can be questioned in unsafe situations with simultaneous motorized road traffic or depending on the road profile. Fixed-gear cycling has previously been studied from a sociological and a physiological point of view (Babault et al., 2018, 2020; Eichler, 2017). However, the neuromuscular activity of fixed-gear cycling has not been evaluated in ecological settings. Given the development and the legality question of this urban practice (Resor, 2011), exploring unsafe situations could help individuals with a safer practice. Accordingly, the present case study aimed to determine muscle electromyographic activity during two decelerating strategies used by a single fixed-gear cyclist to control the bicycle speed while riding downhill a mountain.

## Methods

### Participant

A single fixed-gear cyclist was included. The volunteer (30 years, 179 cm of body height and 66 kg of body mass) was a former international ironman triathlete. The average annual cycling volume was 10,000 km with 90% with a fixed-gear bicycle. The volunteer used his own fixed-gear bicycle (Look 875 Madison, Nevers, France). The bicycle was 6.2 kg with a 45 x 18 gear development, automatic pedals and fully brakeless. The cyclist either used isometric contractions on the pedals/crank to block the rear wheel (i.e., skidding technique) or eccentric contractions to slow the pedals/crank/wheels rotations. The cyclist reported the right as being his preferred side (i.e., the left pedal on the front) for skidding braking strategies. The volunteer was fully informed and signed a written informed consent form before participation. This study was conducted according to the Declaration of Helsinki and approved by the local Institutional Review Board (CERSTAPS, approval code: IRB00012476-2023-17-02-230; approval date: 17 February 2023).

### Design and Procedure

**Figure 1:**
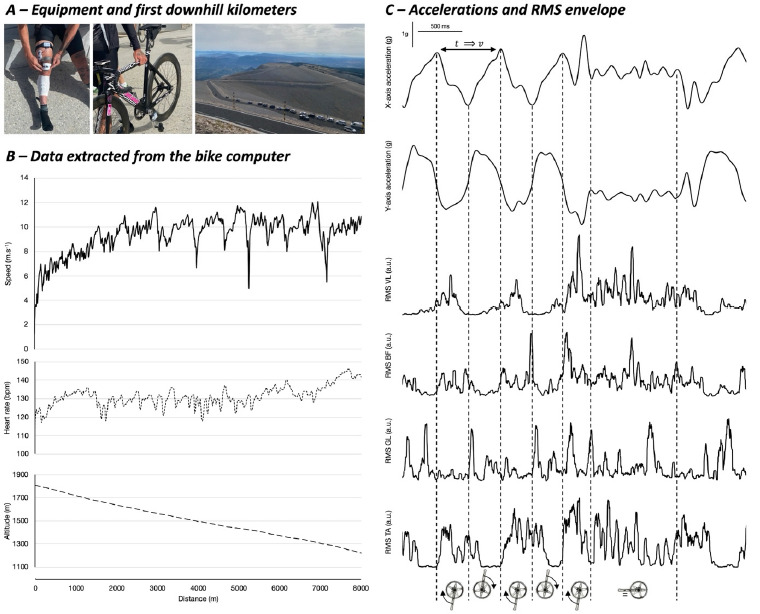
A. Pictures of the volunteer with electromyography, the fixed-gear bicycle with the accelerometer over the left crank and the first kilometers of the downhill ride. B. Speed, heart rate and altitude obtained from the bike computer. The first 8 km are shown to illustrate the significant speed variations related to both eccentric and isometric contractions (skids). C. X and Y axe accelerations were synchronized with the root mean square (RMS) envelope of VL (vastus lateralis), BF (biceps femoris), GL (gastrocnemius lateralis) and TA (tibialis anterior) muscles. RMS is shown in arbitrary units. Vertical dashed lines indicate the vertical position of the right crank. The subsequent direction of the rotation is indicated with the arrow. Note that the last crank picture illustrates an isometric contraction corresponding to a skid. The double arrow represents the duration (*t*) of a complete pedaling cycle that permits the calculation of the bicycle velocity (*v*).

The volunteer was first equipped with surface electromyographic electrodes (Bionomadix, Biopac System, Santa Barbara, USA). After the skin was shaved, abraded and cleaned with alcohol, four pairs of silver-chloride electrodes were positioned on the right side over the vastus lateralis (VL), the long head of the biceps femoris (BF), the gastrocnemius lateralis (GL) and the tibialis anterior (TA) muscles according to SENIAM recommendations. Electrodes were of a 10-mm diameter with a 20-mm interelectrode distance. Low impedance (<2,000 W) was first obtained by shaving, abrading and cleansing with alcohol. A reference electrode was pasted over the right patella. Signals were registered at a 2,000 Hz sampling frequency, bandpass filtered (20–500Hz) and the root mean square envelope (RMS) was subsequently calculated over a 25-ms moving window (Hug et al., 2008). RMS values were normalized according to maximal values obtained during the whole downhill ride. A tri-axial accelerometer (Bionomadix, Biopac System, Santa Barbara, USA) was positioned over the left crank to register the bicycle pedaling frequency and identify pedaling patterns during isometric or eccentric actions (Figure 1). Accelerations were measured at a 200-Hz sampling frequency and lowpass filtered (10 Hz). The heart rate, bicycle velocity and GPS traces were registered using a Garmin Edge 1030 bike computer (Garmin LTD, Olathe, USA).

After being prepared, the rider had to cycle downhill on an asphalt road at his own preferred speed. The road, often used during international cycling events, was the Mont Ventoux: summit to Bedoin (France). The total length was 21.1 km with a 1,503-m negative altitude difference. Measurements were conducted in August on a sunny day (24°C) without any significant wind. The rider was instructed to ride safely at his preferred speed. This study aimed to explore braking strategies without any potential fatigue. For this reason, only some cycles within the first eight kilometers were considered for analyses. First, accelerations were used to identify the pedaling pattern (i.e., eccentric or isometric) and determine the pedaling cycle that was defined between two consecutive top dead center positions (0° = highest pedal position). For a given cycle, we extracted the total cycle duration, the mean and peak RMS values as well as the percentage of the cycle corresponding to the RMS onset and activation duration (> 5% of the maximal values). A 50-ms electromechanical delay was applied (Hug et al., 2008). The preceding and subsequent cycle duration was also measured to determine the bicycle speed variation.

## Results

The average bicycle speed and the heart rate during the 38-min 14-s total downhill ride were 33.1 ± 1.5 km∙h^−1^ (range: [14.3;43.4], CV (coefficient of variation): 16.4%) and 132 ± 6 bpm (range: [118;152], CV: 4.6%), respectively. A total of 90 eccentric cycles (on average 10 consecutive cycles every kilometer) were analyzed. The mean duration of eccentric cycles was 536 ± 51 ms (range: [473;725], CV: 9.6%). The cycle duration variation (between the preceding and following cycle) was −1.4 ± 3.6 % (range: [−10.2;12.3], CV: 261%).

Nineteen isometric contractions were analyzed. On average, the duration of isometric cycles was 1,092 ± 281 ms (range: [731;1,788], CV: 25.8%). The purely isometric phase was 570 ± 241 ms (range: [257;1,088], CV: 42.4%) which was 7.9 ± 39.9% longer than the dynamic phase (range: [−52.1;72.1], CV: 505.2%). The cycle duration variation after isometric braking cycles was 13.0 ± 4.7% (range: [6.7;22.6], CV: 36%).

**Figure 2:**
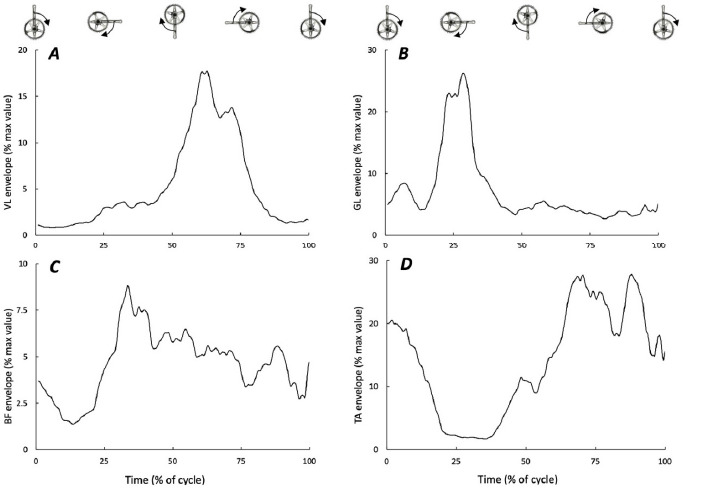
Typical root mean square (RMS) envelopes for the four muscles are in panels A (VL, vastus lateralis), B (GL, gastrocnemius lateralis), C (BF, biceps femoris), and D (TA, tibialis anterior). Envelopes were obtained by calculating the average RMS of the normalized cycle over 10 consecutive eccentric cycles. RMS values are given as % of the maximal values obtained during the whole downhill ride.

RMS amplitudes were lower during eccentric cycles as compared to isometric ones for most muscles (Table 1). The higher percentage difference as compared to average CV for the VL revealed significant differences between eccentric and isometric cycles (Table 1) (Merino-Muñoz et al., 2020). The duration of muscle activation represented half of the eccentric cycle for the GL muscle and more than 60% for VL, TA and BF muscles (see Figure 2 for typical RMS envelope traces). The onset of muscle activity was 10% before the top dead center (0°) for the GL, 13% after this position for VL and BF muscles and 35% for the TA. During isometric cycles, all muscles were activated during the first quarter of the cycle and with activation lasting longer than 80% of the total cycle. The duration (start and length) of muscle activation during eccentric and isometric cycles was different for VL and GL muscles (Table 1).

**Table 1:** EMG activity of the different lower limb muscles.

Cyc.	Var.	Vastus Lateralis				Biceps Femoris				Gastrocnemius Lateralis				Tibialis Anterior				
		Timing (%)		Activity (%)		Timing (%)		Activity (%)		Timing (%)		Activity (%)		Timing (%)		Activity (%)		
		Start	Dur.	Mean	Peak	Start	Dur.	Mean	Peak	Start	Dur.	Mean	Peak	Start	Dur.	Mean	Peak
ECC	Mean	13.7	64.9	4.3	16.4	13.2	73.5	5.4	16.7	−10.1	49.7	8.0	36.6	35.9	72.9	12.3	34.9
	SD	8.9	9.5	3.5	8.4	9.5	12.1	6.5	9.7	5.7	9.9	2.5	16.1	8.9	7.7	3.3	7.9
	Min	−7.2	44.5	1.0	2.2	0.8	43.9	1.9	6.1	−25.6	32.5	2.8	2.8	22.9	27.4	3.7	17.6
	Max	44.2	85.7	33.2	44.9	35.9	103.0	49.0	89.7	0.6	97.7	18.5	75.8	78.7	84.9	23.8	57.0
	CV	65.1	14.6	82.3	51.6	71.7	16.5	119.7	58.1	56.5	19.9	31.7	44.0	24.7	10.6	26.6	22.8
ISO	Mean	5.2	83.2	17.9	60.9	10.1	85.5	7.4	25.3	20.1	83.7	10.3	48.1	15.4	85.6	19.4	57.8
	SD	3.1	20.8	2.6	13.0	3.2	8.2	1.0	2.9	3.8	13.2	2.5	14.6	3.8	5.3	5.8	18.3
	Min	0.2	0.1	11.1	41.6	5.3	61.2	5.2	20.5	14.8	60.2	5.7	23.4	8.2	76.1	1.8	6.4
	Max	11.9	104.8	23.1	90.1	15.9	100.0	9.7	30.0	27.5	100.0	15.0	88.2	24.9	92.7	30.2	98.5
	CV	58.2	25.0	14.4	21.4	31.9	9.5	13.5	11.3	18.7	15.7	24.4	30.4	24.5	6.2	29.7	31.7
ECC-ISO (%Diff.)		89.2	−25	−122	−115	26.8	−15	−31	−41	−601	−51	−25	−27	79.9	−16	−44	−49
ECC-ISO (CVa)		72.1	20.7	92.8	80.1	69.4	16.4	103.3	52.1	263.2	29.9	31.9	42.6	35.0	11.8	34.4	35.1

Cyc.: Cycle; Var.: variable; Dur.: duration; ECC: eccentric pedaling cycle; ISO: isometric cycle; SD: standard deviation; Min: minimum; Max: maximum; CV: coefficient of variation; Start: onset of EMG activity; Length: duration of the EMG activity; ECC-ISO (%Diff.): percentage difference between eccentric and isometric mean values; ECC-ISO (CVa): average coefficient of variation between eccentric and isometric contractions. Duration times are presented as % of the total cycle duration and EMG activity as % of the maximal activity during the whole downhill ride.

## Discussion

The present case study was conducted to initiate exploring braking techniques in fixed-gear brakeless cycling and eccentric cycling under ecological conditions. In order to control the downhill bicycle velocity, the rider used two different strategies. The rider mainly used the eccentric pedaling strategy to efficiently control the bicycle velocity (like an even pacing strategy). The eccentric cycling pattern was significantly different than the concentric one, more particularly for VL and BF muscles (Green et al., 2018; Hug et al., 2008). For instance, while the VL is activated around the top dead center during concentric cycling in laboratory settings (Green et al., 2018; Hug et al., 2008), it is mostly activated after the bottom dead center during our eccentric downhill cycling exercise. In addition, a longer BF activation appeared as compared to concentric pedaling. Obviously, even in laboratory settings, muscle activation is always lower in eccentric as compared to concentric pedaling (Green et al., 2018; Peñailillo et al., 2017) due to the different neural drive between these two action modes (Babault et al., 2001). When comparing to eccentric cycling literature, our results revealed almost similar electromyographic patterns to those previously described (Peñailillo et al., 2017; Walsh et al., 2022; Tortu and Deliceoglu, 2024). For instance, VL activation started slightly after the bottom dead center. However, one should note that these various electromyographic patterns might imply very different muscle-tendon behaviors. Indeed, in laboratory/rehabilitation settings, eccentric cycling is performed backward (anti-clockwise direction) using semi-recumbent ergocycles, while, in our case (ecological condition in downhill situations), the rider used eccentric contractions with clockwise pedaling in a seated position. These discrepancies would mostly affect biarticular muscles such as the rectus femoris that would act with shortened lengths under fixed-gear conditions. In addition, muscles are activated for a longer time than a half cycle with a significant temporal range of activation. Such findings are concomitant with significant ranges of activation for all four muscles. It illustrates various activation strategies mostly depending on velocity control.

The second braking strategy was to apply long and intensive isometric contractions of the main lower limb muscles to block the rear wheel. Depending on the road profile and velocity, this technique can be repeated twice. Such a skid technique used friction forces for larger speed reductions than the eccentric strategy. Considering some potential hazardous loss of bike control or tire puncture, this technique is less frequently used. As compared to eccentric contractions, all muscles are activated approximately at the same time (slightly later for gastrocnemius medialis muscle) during more than 80% of the entire cycle. Muscle activation was larger than under eccentric conditions, more particularly for the VL. These simultaneous maximal contractions are associated with a totally different biomechanical posture. Indeed, to favor skidding and lighten the rear wheel, riders reorganize weight distribution forward, lifting from the saddle and pushing the handlebar. An important activation of upper body muscles is therefore required and could influence fatigue or modify ventilation (Lechauve et al., 2014).

## Conclusions

The results of this case study suggest specific muscle activation while riding a fixed-gear bicycle downhill depending on the deceleration strategies and as compared to the generally investigated laboratory eccentric cycling. Two main questions may arise from these general observations. First, for safety rides, one should consider exploring further the best strategies to decelerate a fixed-gear bicycle while riding downhill or on flat roads. Second, one can question whether eccentric cycling prescribed in rehabilitation settings can be used under seated conditions (rather than the predominant semi-recumbent position) while pedaling backward but also forward. Exercising in both directions would allow for various muscle solicitations and subsequent neuromuscular adaptations.
